# Supplementary material for: COVID-19 vaccination data management and visualization systems for improved decision-making: Lessons learnt from Africa CDC Saving Lives and Livelihoods program
Source: PLOS Digit Health. 2025 Dec 11;4(12):e0000782. doi: 10.1371/journal.pdig.0000782 (PMC12697957; doi:10.1371/journal.pdig.0000782)
Supplement: Attachment — Submitted filename: 2025_PLOS_Covid.pdf [file pdig.0000782.s004.pdf]

Review of “COVID-19 Vaccination Data Management and Visualization Systems for Improved Decision-Making: Lessons Learnt from Africa CDC”

This paper provides a valuable and positive account of Africa CDC’s efforts to develop and implement a continental vaccination data management and visualization system during the COVID-19 pandemic. It documents a successful initiative that had tangible impact on coordination, reporting, and decision-making, and it offers lessons that will be relevant for digital health practitioners, policymakers, and researchers worldwide.

Strengths: The topic is timely and important, demonstrating the feasibility of large-scale digital infrastructure for public health in Africa. The lessons drawn from the Africa CDC experience provide actionable insights for strengthening preparedness and response in future health emergencies. Further, the manuscript is written in a way that can be understood by both specialists and non-specialists, broadening its reach.

Areas for Improvement: While the paper succeeds in documenting a successful project, it currently reads more as a success story than a critical academic study. The discussion often feels too general, lacking sufficient depth on challenges and evidence of impact. To strengthen its contribution, the following points should be addressed:

- **Evidence of Impact:** The current version is largely descriptive. Including quantitative metrics (e.g., data completeness, timeliness, accuracy) and case studies of how dashboards influenced policy would provide stronger evidence.
  Comparative analysis of vaccination outcomes with and without the system would add weight to claims of improved decision-making.
- **Clarity and Vagueness:** Descriptions of the system and its effects remain broad. Specific examples–such as performance indicators, user feedback, or schematic diagrams of the data pipeline—would add clarity and rigor without reducing accessibility.
- **Limitations and Challenges:** A dedicated section on barriers would provide balance. This could include political and logistical hurdles, scalability concerns, and the “last mile” issues of data quality from local facilities where internet connectivity, staffing, and reliance on paper-based records remain challenges.
- **Presentation**: Minor typographical issues should be corrected (e.g., “COVID-10 vaccination data,” “apps.apps”), and references need formatting consistency.

A further concern is the reliance on a single platform. While DHIS2 was a pragmatic and widely used choice, it also brings risks—such as functionality gaps, limited flexibility, or technical debt from customisation—that are not addressed in the paper. In addition, the authors should clarify how accessible the system is from an open science perspective.

In summary, this is a timely and worthwhile paper that makes an important contribution by documenting a successful regional digital health initiative. With revisions to reduce vagueness, provide firmer evidence of impact, and present a more critical analysis of challenges and platform dependencies, the paper would be substantially strengthened and well suited for publication in *PLOS Digital Health*.
