# Supplementary material for: COVID-19 vaccination data management and visualization systems for improved decision-making: Lessons learnt from Africa CDC Saving Lives and Livelihoods program
Source: PLOS Digit Health. 2025 Dec 11;4(12):e0000782. doi: 10.1371/journal.pdig.0000782 (PMC12697957; doi:10.1371/journal.pdig.0000782)
Supplement: Attachment — Submitted filename: Response to Reviewers.docx [file pdig.0000782.s005.docx]

**Response to Reviewers


Journal Requirements:**

1. We note that your Data Availability Statement is currently as follows: All data are in the manuscript files.
 
 Please confirm at this time whether or not your submission contains all raw data required to replicate the results of your study. Authors must share the “minimal data set” for their submission. PLOS defines the minimal data set to consist of the data required to replicate all study findings reported in the article, as well as related metadata and methods (<https://journals.plos.org/plosone/s/data-availability#loc-minimal-data-set-definition>).
 
 For example, authors should submit the following data:
 
 - The values behind the means, standard deviations and other measures reported;
 - The values used to build graphs;
 - The points extracted from images for analysis.
 
 Authors do not need to submit their entire data set if only a portion of the data was used in the reported study.
 
 If your submission does not contain these data, please either upload them as Supporting Information files or deposit them to a stable, public repository and provide us with the relevant URLs, DOIs, or accession numbers. For a list of recommended repositories, please see <https://journals.plos.org/plosone/s/recommended-repositories>.
 
 If there are ethical or legal restrictions on sharing a de-identified data set, please explain them in detail (e.g., data contain potentially sensitive information, data are owned by a third-party organization, etc.) and who has imposed them (e.g., an ethics committee). Please also provide contact information for a data access committee, ethics committee, or other institutional body to which data requests may be sent. If data are owned by a third party, please indicate how others may request data access.


**Authors' Response –** We appreciate the reviewers' valid point. The manuscript primarily focuses on sharing lessons learned from the implementation process, with minimal use of performance or research-specific data. To support the few data points referenced, we have provided an accompanying Excel sheet detailing the relevant information.

2. Please provide separate figure files in .tif or .eps format.

For more information about figure files please see our guidelines: 
<https://journals.plos.org/digitalhealth/s/figures> 
<https://journals.plos.org/digitalhealth/s/figures#loc-file-requirements>

**Authors’ Response –** We thank you for the feedback. All five figures are attached in .tif format.

3. Please upload a copy of Figure 1, 2, 3, 4, 5 which you refer to in your text on page 9, 10, 12, 14, 15. Or, if the figure is no longer to be included as part of the submission please remove all reference to it within the text.

**Authors’ Response** – Many thanks. Now, all five figures are attached as .tif format.

4. Some material included in your submission may be copyrighted. According to PLOS’s copyright policy, authors who use figures or other material (e.g., graphics, clipart, maps) from another author or copyright holder must demonstrate or obtain permission to publish this material under the Creative Commons Attribution 4.0 International (CC BY 4.0) License used by PLOS journals. Please closely review the details of PLOS’s copyright requirements here: PLOS Licenses and Copyright. If you need to request permissions from a copyright holder, you may use PLOS's Copyright Content Permission form.

Please respond directly to this email or email the journal office and provide any known details concerning your material's license terms and permissions required for reuse, even if you have not yet obtained copyright permissions or are unsure of your material's copyright compatibility.

Potential Copyright Issues:
Figures 2 and 4: please (a) provide a direct link to the base layer of the map (i.e., the country or region border shape) and ensure this is also included in the figure legend; and (b) provide a link to the terms of use / license information for the base layer image or shapefile. We cannot publish proprietary or copyrighted maps (e.g. Google Maps, Mapquest) and the terms of use for your map base layer must be compatible with our CC-BY 4.0 license.

Note: if you created the map in a software program like R or ArcGIS, please locate and indicate the source of the basemap shapefile onto which data has been plotted.

If your map was obtained from a copyrighted source please amend the figure so that the base map used is from an openly available source. Alternatively, please provide explicit written permission from the copyright holder granting you the right to publish the material under our CC-BY 4.0 license.

Please note that the following CC BY licenses are compatible with PLOS license: CC BY 4.0, CC BY 2.0 and CC BY 3.0, meanwhile such licenses as CC BY-ND 3.0 and others are not compatible due to additional restrictions.

If you are unsure whether you can use a map or not, please do reach out and we will be able to help you. The following websites are good examples of where you can source open access or public domain maps: 
* U.S. Geological Survey (USGS) - All maps are in the public domain. (<http://www.usgs.gov>) 
* PlaniGlobe - All maps are published under a Creative Commons license so please cite “PlaniGlobe, <http://www.planiglobe.com>, CC BY 2.0” in the image credit after the caption. (<http://www.planiglobe.com/?lang=enl>) 
* Natural Earth - All maps are public domain. (<http://www.naturalearthdata.com/about/terms-of-use/>)

**Author's Response:**

The map was generated directly from the DHIS2 platform using its built-in analytics and mapping functionalities. As a registered user of DHIS2, we accessed this visualization through the platform’s dashboard, and it represents data layers configured within our organization instance.

According to the DHIS2 documentation on maps, DHIS2 uses a combination of open-source and external map tile providers. The default base maps in DHIS2 (e.g., OpenStreetMap) are publicly available under permissive licenses, and DHIS2 supports exporting such maps for internal use, reporting, and publication, provided attribution is maintained where applicable.

The map included in the manuscript does not modify the base tiles and serves solely for illustrative purposes. Nevertheless, we have added the appropriate attribution in the manuscript’s figure caption in line with the DHIS2 documentation.

**OpenStreetMap Copyright and License:** https://www.openstreetmap.org/copyright
**DHIS2 documentation:** <https://docs.dhis2.org/en/topics/tutorials/google-earth-engine-sign-up.html>

**Additional Editor Comments**

**Reviewers' Comments:**

Reviewer #1: The paper details the development of a continental COVID-19 vaccination tracking system by Africa CDC and HISP-SA using DHIS2. The system enables real-time monitoring of vaccine distribution, with 300 users accessing role-based dashboards. It provides high-level summaries for leadership and detailed data for programmatic users, supporting informed decision-making. As COVID-19 remains a concern, the system continues to track vaccine uptake and related metrics. Its success offers a model for managing public health data on a large scale.

Need to check on data availability as there was no attachment or link seen.

**Authors Response:**

- The manuscript primarily focuses on sharing lessons learned from the implementation process, with minimal use of performance or research-specific data. To support the few data points referenced, we have provided an accompanying Excel sheet detailing the relevant information.

Reviewer #1:

Some sentences are overly complex and could be revised for clarity. There are minor grammatical issues and typos that should be corrected. The use of technical terms should be consistent and well-defined to ensure clarity for a broad readership.

**Authors Response:**

We thank you for the important feedback here. We have exhaustively addressed the points in the document where some of the parts seem complex.

The research topic is highly relevant and timely and the manuscript is generally well-structured.

Reviewer #2: This paper is timely and a good summary of the seminal efforts that went to into digitizing the support required to power vaccination delivery and results monitoring. Having said that, the manuscript would be better if certain additional details were provided. This will be esp. beneficial for the readers and other stakeholders in LMIC settings who would like to replicate such efforts in their public health delivery programs. Listing them below:

1. Please provide some specific context of the challenges encountered by the stakeholders before the data management system and dashboard were in place. E.g. it took the joint steering committee 1 month to receive upto date information to make key decisions, before digitization and near real time updates.

**Authors Response:**

Many thanks for the excellent insight here. This is addressed as “At the begining of the COVID-19 vaccination rollout, Africa CDC encountered significant challenges in coordinating and monitoring efforts across Member States due to the absence of a centralized, real-time data system. Data on vaccine distribution, uptake, and stock levels were often fragmented, delayed, or inconsistently reported, limiting Africa CDC’s ability to track progress, identify gaps, and provide timely technical support. The lack of standardized digital tools also made it difficult to consolidate information across countries, hindering strategic decision-making and efficient allocation of vaccines and resources. These challenges underscored the urgent need for a unified digital platform to enable accurate, timely, and actionable insights for effective pandemic response” in the introduction section. **[Page 4, Paragraph 1, line 3 -11]**

2. How long did it take for the data management system to go live and become functional? from concept to production?

**Authors Response:**

Many thanks for the important comment here. We revised the document accordingly **[Page 6, Paragraph 1, line 2-10]**
3. What were the reasons for choosing the DHIS2 platform? E.g. its open source, handles multiple data sources, works in offline settings, can be customized and allows building apps on it.
**Authors Response:**
Many thanks for the important comment here. We revised the document accordingly.

This is adressed in the manuscript as “ DHIS2 was selected as the preferred platform due to its open-source nature, flexibility, and widespread use across African countries for routine health data reporting. Its adaptability to diverse programmatic needs, strong global community, and the presence of local technical expertise in many Member States facilitated rapid adoption and localization. By leveraging an already familiar system, Africa CDC ensured alignment with existing national health information infrastructures, promoted interoperability, and enabled efficient scale-up of digital solutions across the continent.” under Contents and process of the dashboard development. **[Page 6, Paragraph 1, line 2-10]**

4. what were the highest level KPIs in tracking the buildout and rollout of this data management system? was it done in phases? if so, how was it done?

**Authors Response:** Great insight. This is addressed in the manuscript “ The dashboard was designed in phases, beginning with the design of data collection forms and reporting datasets, followed by the creation of data visualization dashboards, and culminating in modules for data sharing, interpretation, and use” under Contents and process of the dashboard development. **[Page 6, Paragraph 1, line 8-10]**

5. One of the key takeaways on the paper is the governance structure about the 4 decision making bodies- joint steering committee, program management unit, vaccine taskforce and partnership management. Please provide a schematic diagram for these four bodies, key decisions each makes and the data sources + information needed to execute their work. It will be easier for the readers to grasp it visually.

**Authors Response:**

We thank the reviewers for the valid suggestion.

6. What if any, would be best practice recommendations and practical tips for agencies in other LMIC settings to build similar digital infrastructure for their public health initiatives.

**Authors Response:**

We have addressed this in the conclusion section of the manuscript as “ Translating this framework of digital immunization dashboards to other public health areas—while leveraging existing digital infrastructure—can enable more efficient use of resources, promote interoperability and standardization across countries, particularly in low- and middle-income countries (LMICs), and ultimately enhance health equity and advance digital health initiatives across the continent” . **[Page 19, paragraph 1, line 2-6]**
