# Supplementary material for: COVID-19 vaccination data management and visualization systems for improved decision-making: Lessons learnt from Africa CDC Saving Lives and Livelihoods program
Source: PLOS Digit Health. 2025 Dec 11;4(12):e0000782. doi: 10.1371/journal.pdig.0000782 (PMC12697957; doi:10.1371/journal.pdig.0000782)
Supplement: Attachment — Submitted filename: Response_to_Reviewers_auresp_2.docx [file pdig.0000782.s006.docx]

**Response to Reviewers and Editor**

- **Evidence of Impact:** The current version is largely descriptive. Including quantitative metrics (e.g., data completeness, timeliness, accuracy) and case studies of how dashboards influenced policy would provide stronger evidence.

**Author response:** *Thank you for this insightful comment. In response, we have incorporated quantitative metrics to demonstrate the system’s impact in terms of vaccine dose management, resource allocation, training, and vaccination. These additions are presented at the beginning of the Results section of the manuscript..*

- **Clarity and Vagueness:** Descriptions of the system and its effects remain broad. Specific examples–such as performance indicators, user feedback, or schematic diagrams of the data pipeline—would add clarity and rigor without reducing accessibility.

  **Author response:** *Thank you for this valuable comment. To enhance clarity, we have added a schematic diagram (Fig. 1) illustrating the summary data flow, as well as a summary of user feedback in the Monitoring and Evaluation (M&E) dashboard section. A full list of dashboard metrics/performance indicators is shown in Table 1 of the manuscript.*
- **Limitations and Challenges:** A dedicated section on barriers would provide balance. This could include political and logistical hurdles, scalability concerns, and the “last mile” issues of data quality from local facilities where internet connectivity, staffing, and reliance on paper-based records remain challenges

**Author Response:** *Thank you for the comment.* *There was a dedicated section in the manuscript on challenges and limitation. We separated and expanded the two sections for more clarity and avoid ambiguity (Both can be found at the end of the manuscript between discussion and conclusion section)*

- **Presentation**: Minor typographical issues should be corrected (e.g., “COVID-10 vaccination data “ apps.apps”, and references need formatting consistency

**Author Response:** *Thank you for this comment. This is addressed accordingly*

- A further concern is the reliance on a single platform. While DHIS2 was a pragmatic and widely used choice, it also brings risks—such as functionality gaps, limited flexibility, or technical debt from customization—that are not addressed in the paper. In addition, the authors should clarify how accessible the system is from an open science perspective.

**Author Response:** *Thank you for the comment***. *The limitation section addressed this concern “No*** *cost-effectiveness comparison with other digital platforms is presented, which could enhance contextual understanding”.

The reason for selecting DHIS2 for this project was well described in the* ***Contents and process of the dashboard development*** *section of the manuscript where DHIS2 is identified as the common and widely implemented system in Africa which makes it the better choice for continental and emergency impelemntation during the time.*
 .
“…..We used an open-source web-based software platform called DHIS2 software (18) for the dashboard development. DHIS2 was selected as the preferred platform due to its open-source nature, flexibility, and widespread use across African countries for routine health data reporting. Its adaptability to diverse programmatic needs, strong global community, and the presence of local technical expertise in many Member States facilitated rapid adoption and localization…..”

*Regarding open science and accessibility, the DHIS2 platform’s open-source architecture and role-based access controls provide data security while enabling authorized users across various organizational levels to view and interact with dashboards tailored to their roles. The manuscript clarifies data access is managed through authenticated accounts with defined privileges, balancing transparency and data confidentiality* ***[Methods—Access to online data and validation process]****. This approach aligns with open science principles by supporting data-driven decision-making while safeguarding sensitive information.*
